# Elevated BMI is considerably associated with IDD rather than polymorphic variations in interleukin-1 and vitamin D receptor genes: A case-control study

**DOI:** 10.5937/jomb0-26367

**Published:** 2021-03-12

**Authors:** Mazhar Salim Al-Zoubi, Osama Otoum, Mohammed Alsmadi, Riyadh Muhaidat, Ahmed Albdour, Ziyad Mohaidat, Manal Issam Abu Alarjah, Raed M. Al-Zoubi, Khalid M. Al-Batayneh

**Affiliations:** 1 Yarmouk University, Faculty of Medicine, Department of Basic Medical Sciences, Irbid, Jordan; 2 Yarmouk University, Faculty of Science, Department of Biological Sciences, Irbid, Jordan; 3 King Hussein Medical Centre, Royal Medical Services, Amman, Jordan; 4 Jordan University of Science and Technology, Faculty of Medicine, Irbid, Jordan; 5 Jordan University of Science & Technology, Department of Chemistry, Irbid, Jordan

**Keywords:** IDD, VDR, IL-1α, IL-1β, rs1800587, rs1143634, rs2228570, rs731236, IDD, VDR, IL-1α, IL-1β, rs1800587, rs1143634, rs2228570, rs731236

## Abstract

**Background:**

Intervertebral disc degeneration (IDD) is a musculoskeletal disorder and one of the major causes of low back pain leading to the disability with high economic repercussions worldwide. This study applied the candidategene approach to investigate the potential association of selected polymorphisms with IDD development in a Jordanian population.

**Methods:**

MRI-diagnosed IDD patients (N=155) and asymptomatic individuals as a control group (N=55). Whole blood samples for four variants in three genes (*rs1800587* of *IL-1α*, *rs1143634* of *IL-1β* and *rs2228570* and *rs731236* of *VDR*) were genotyped by PCR-RFLP.

**Results:**

There was no significant association between the studied polymorphisms or their allelic frequency and the occurrence of IDD. However, the cohort presented a significant reverse association between *rs1143634 *C > T of the *IL-1β* gene and the occurrence of IDD (p<0.0001). In addition, BMI showed a significant association with the IDD in the study population (p<0.005). The current study was conceptualized based on the candidate-gene approach to investigate the role of inflammatory and metabolic genes, IL and VDR, respectively, in the occurrence of IDD.

**Conclusions:**

While the data presented in this study showed that polymorphisms in these genes were not associated with IDD of the cohort investigated, elevated BMI, as a measure of obesity, is strongly associated with IDD. Investigating potential roles of other structural genes, such as col-IX and aggrecan (ACAN), in IDD and considering a GWAS to elucidate a genomically global look at the basis of IDD development would be of considerable impact on our understanding of IDD.

## Abstract (sr)

**Uvod:**

Degeneracija intervertebralnog diska (IDD) je mišićno-koštani poremećaj i jedan od glavnih uzroka bolova u donjem delu leđa koji vodi do invaliditeta i ima velike ekonomske posledice širom sveta. U ovom istraživanju korišćen je pristup određivanja gena kandidata kako bi se istražila potencijalna povezanost odabranih polimorfizama sa razvojem IDD-a u jordanskoj populaciji.

**Metode:**

IDD pacijenti dijagnostikovani putem magnetne rezonantne tomografije (MRI) (N=155) i asimptomatski pojedinci kao kontrolna grupa (N=55). Određen je genotip celokupnih uzoraka krvi za četiri varijante u tri gena (*rs1800587* u *IL-1α*,* rs1143634* u *IL-1β* i r*s2228570* i *rs731236* u *VD*R) pomoću PCR-RFLP.

**Rezultati:**

Nije bilo značajne povezanosti između proučavanih polimorfizama ili njihove učestalosti alela i pojave IDD-a. Međutim, kohorta je pokazala značajnu obrnutu povezanost između *rs1143634* C>T koji pripada genu *IL-1β*, i pojave IDD-a (p<0,0001). Pored toga, BMI je pokazao značajnu povezanost sa IDD-om u ispitivanoj populaciji (p<0,005). Trenutno istraživanje je koncipirano na osnovu pristupa određivanja gena kandidata kako bi se istražila uloga upalnih (IL) i metaboličkih (VDR) gena pri pojavi IDD-a.

**Zaključak:**

Dok su podaci predstavljeni u ovom istraživanju pokazali da polimorfizmi u ovim genima nisu povezani sa IDD-om kohorte koja je istra`ivana, povišen BMI, kao mera gojaznosti, snažno je povezan sa IDD-om. Ispitivanje potencijalnih uloga drugih strukturalnih gena, poput col-IX i agrekana (ACAN) u IDD-u, i razmatranje GWAS-a za razjašnjenje genomski globalnog pogleda na osnovu razvoja IDD-a, imalo bi značajan uticaj na naše razumevanje IDD-a.

## Introduction

Intervertebral disc degeneration (IDD) is a chronic musculoskeletal disease characterized by a gradual loss of water and proteoglycans (PGs) from the nucleus pulposus with a high incidence of asymptomatic cases. Habitually, IDD is an unavoidable outcome of ageing; degeneration begins as early as the second decade of life. IDD affects about 84% of people worldwide with direct and indirect economic influence [1] [2]. At the molecular level, IDDs are intimately associated with a sequence of biochemical and morphological changes in the discs that alter the biomechanical characters of the vertebrae [3] [4]. The collagen network formed mostly of type I and type II collagen fibrils provides radially distributed tensile strength to the disc and anchors the tissue to the bone [5]. As age increases, type II collagen production decreases, whereas type I collagen synthesis increases, leading to less compliant type I collagen. Consequently, the ratio and the relative distribution of type I and type II collagen in the outer annulus and a decrease in collagen cross-links make the annulus more susceptible to mechanical failure [6].

The complex nature of IDD development has been conceived by the interaction of unfavourable inheritance with many physical factors, such as occupation and workload, and other biological factors such as age, senescence, apoptosis, extracellular matrix composition with water content, and certain enzymatic activity levels. With all that taken together, disc impairment gradually increases to irreversible degeneration under certain sudden events or injuries [7]. Nonetheless, genetic factors have been described as major contributors to IDD, accounting for *~*70% of the variability in disc degeneration among identical twins [8]. Recent studies confirmed IDD is associated with variable polymorphic gene families with IDD, especially those of extracellular matrix [9]. Several structural, metabolic, inflammatory, and other genes have been found to play roles in developing IDD, and have been investigated in various populations, including Finnish, Japanese, and Chinese [10] [11] [12].

Allelic variation of the *VDR* gene has been found to be causative of musculoskeletal diseases, such as osteoarthritis [13] and osteoporosis [14], suggesting that *VDR* gene polymorphisms may underlie the development of IDD. For instance, *rs731236* and *rs2228570 VDR* and *IL-1* gene polymorphisms have frequently been investigated for their potential role in IDD [2] [4]. *IL-1α* gene promoter holds a C > T polymorphism (*rs1800587*) that is associated with the increased transcriptional activity of the gene when compared with the CC genotype [4]. In addition, the *rs1143634* variant (C > T) in exon 5 of the *-1* gene has been found to be associated with an increase in *IL-1β* production levels [15].

The contribution of genetic factors to IDD development has been well studied in many populations such as Finnish, Spanish, Chinese, and Japanese, and, to our knowledge, none has so far been carried out in Jordan. Therefore, the current study aimed at examining selected polymorphisms *rs1800587* of *IL-1α*, *rs1143634* of *IL-1β* and *rs2228570* and *rs731236* of *VDR* genes and evaluating their potential association with IDD in the Jordanian population.

## Materials and Methods

### Patients and Sample collection

Fresh whole blood samples for DNA extraction were collected from 210 individuals, 155 samples from IDD patients diagnosed by MRI at Prince Rashed Bin Al-Hasan Military Hospital, and the remaining 55 samples were collected from normal people (having no visual or apparent symptoms). Informed consent forms, as well as questionnaires, were taken after obtaining ethical approval from the Research Ethics Committee at Yarmouk University. Briefly, 4 mL of whole blood was collected in EDTA coated vacutainer tubes, and stored at 4°C until use.

#### DNA amplification and restriction fragment length polymorphism (PCR-RFLP)

The extraction of genomic DNA was carried out using the QIAamp^®^ DNA Blood Mini Kit (QIAGEN, Germany) according to the manufacturer's instructions. Polymorphic variants were analyzed by polymerase chain reaction followed by restriction fragment length polymorphism (PCR-RFLP). A general PCR procedure included three steps: firstly, denaturation at 94°C for 5 min, followed by 35 cycles each cycle consists of denaturation at 94°C for 30 sec, annealing for 30 sec at 55°C and extension for 1 min at 68°C, and finally an extra extension step at 68°C for 5 min. Table 1 summarizes the primer pairs used to amplify the target sequences of the selected genes. The studied genetic variations are summarized in Table 2. A region of 193 bp spanning the *rs1800587* (-899 C > T) polymorphic site at the promoter region of *IL-1α* gene was amplified and digested with a NcoI restriction enzyme (2U for 2h at 37°C) (New England Biolabs Inc., Beverly, MA) [16]. Allele C has a restriction site giving two fragments of 174 and 19 bp, while allele T has no recognition site. Furthermore, a region of 194 bp spanning the *rs1143634* (+3954 C > T) polymorphic site at exon 5 of *IL-1β* gene was amplified and digested with a Taq^α^I restriction enzyme (2U for 2h at 65°C) (New England Biolabs Inc., Beverly, MA). Allele C has two restriction sites giving three fragments of 97, 85, and 12 bp, while allele T has two recognition sites giving two fragments of 182 and 12 bp. PCR product of 265 bp containing the single nucleotide polymorphism (SNP) *rs2228570* (T>C) at the translation initiation codon of exon 2 of VDR gene was digested with a FokI restriction enzyme (1U for 2 h at 37°C) (New England Biolabs Inc., Beverly, MA) [16]. Allele T has a restriction site giving two fragments of 197 and 68 bp, while allele C has no recognition site. Similarly, the 747 bp amplicon spanning the synonymous polymorphism *rs731236* (352 T>C) in exon 9 of the *VDR* gene [17] was cleaved with a Taq^α^I restriction enzyme (2U for 2h at 65°C) (New England Biolabs Inc., Beverly, MA). Allele T showed two fragments of 496 and 251 bp, while allele C generated an additional restriction site giving three fragments of 294, 251, and 201 bp. The resultant fragments from the restriction digestion reaction were detected and analyzed using 3% agarose gel electrophoresis and using a 50 bp DNA marker as a reference.

**Table 1:** Forward and reverse primers for the target sequences.

Genes	Rs #.	Forward primers	Reverse primers	Ref
IL-1α	rs1800587	5’GCATGCCATCACACCTAGTT3’	5’TTACATATGAGCCTTCCATG3’	How et al., 2007
IL-1β	rs1143634	5’CTCAGGTGTCCTCGAAGAAATCAA3’	5’GCTTTTTTGCTGTGAGTCCCG3’	Trevilatto et al., 2011
VDR	rs731236	5’CAGAGCATGGACAGGGAGCAAG3’	5’GCAACTCCTCATGGCTGAGGTCTCA3’	Riggs et al., 2009
VDR	rs2228570	5’AGCTGGCCCTGGCACTGACTCTGCTCT3’	5’ATGGAAACACCTTGCTTCTTCTCCCTC3’	Harris et al., 1997

**Table 2:** Demographic characteristics of subjects included in the study.

Variable	Status	Patients (n=155) n (%)	Control (n=55) n (%)	*P* value
Sex	Male	73(47.3)	24(43.6)	0.7532
	Female	82(52.7)	31(56.4)	
Age	30-40	27(17.1)	10(18.2)	
	41-50	47(30.3)	22(40)	
	51-60	41(26.3)	16(29.1)	
	>60	40(26.3)	7(12.7)	
Age range		30-77	36-77	
Mean Age		52.6	50.6	0.2895
Smoking	Yes	50(32)	14(25)	0.3966
	No	105(68)	41(75)	
BMI	19-25	16(10.4)	10(18.2)	
	26-30	56(36.4)	39(70.9)	
	>30	83(53.2)	6(10.9)	
BMI-total		31	26.5	< 0.005

#### Statistical analysis

The association strength between the target polymorphisms and IDD risk was measured by odds ratio (OR) with a 95% confidence interval (CI) using GraphPad-InStat 3.06 software. The distribution of genotype and allelic frequencies, the risk associated with individual genotype or allele with age, sex, BMI, and smoking distribution in the IDD patients and the control group were compared using t and chi-square tests. P-value is regarded as significant if ≤ 0.05.

## Results

**Figure 1:**
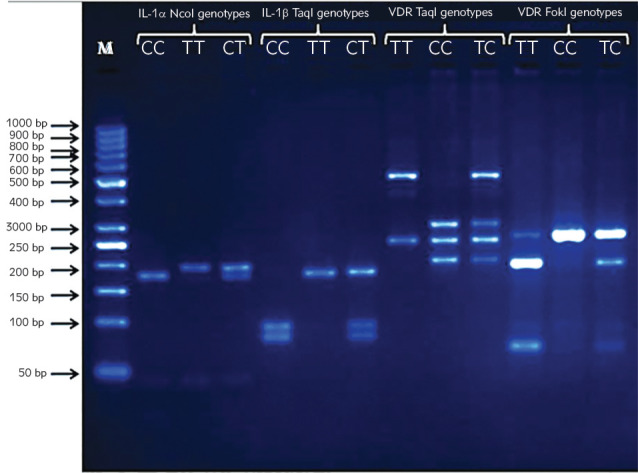
Representative gel for the determination of the polymorphisms *rs1800587, rs1143634, rs731236*, and *rs2228570*, genotypes in twelve subjects are shown. In the first lane, there is a molecular weight DNA ladder (M) for the size estimation of the DNA fragments

Patients with IDD and control subjects with a mean age of 52.6 versus 50.6 years, respectively, were included in the study. In the IDD group, 73 (47.3%) were males compared to the 24 (43.6%) males in the control group. BMI for the IDD and control groups were 31 and 26.5 kg/m^2^, respectively. Therefore, the patients' group fell into the obese class I category while the control group fell into an overweight category, according to the World Health Organization (Table 2). Representative gels for the determination of *rs1800587* of *IL-1α* gene, *rs1143634* of *IL-1β* gene, and *rs2225870* and *rs731236* of VDR gene polymorphisms are shown in Figure 1. *Rs1800587* polymorphism of the *IL-1α* gene showed no significant association with IDD disease (Table 3). The distribution of T risk allele in patients and control was 56.1% versus 56.4% (p=0.976; OR=1.01; 95% CI: 0.5429 to 1.877), genotype frequencies between IDD and control groups were 43.9% versus 43.6% for CC; 14.8% versus 12.7% for TT; and 41.3% versus 43.6% for CT, respectively (p=0.914) (Table 3).

**Table 3:** Genotypes and allelic frequencies of the polymorphisms rs1800587 of IL-1α gene, rs1143634 of IL-1β gene, and rs2228570 and rs731236 of VDR genes for patients and controls

	Genotype	Patients (%)	Control (%)	Statistical analysis	
rs1800587 (IL-1α)	CC	68 (43.9)	24 (43.6)	(χ^2^ = 0.181) (p = 0.914)	
	TT	23 (14.8)	7 (12.7)		
	CT	64 (41.3)	24 (43.6)		
	T Allele frequency	87 (56.1)	31 (56.4)	(OR = 1.01) (95% CI: 0.5429 to 1.877) (p = 0.976)	
rs1143634 (IL-1β)	CC	79 (51)	15 (27.3)	(χ^2^ = 19.253)	
	TT	18 (11.6)	1 (1.8)	(p= <0.0001)	
	CT	58 (37.4)	39 (70.9)		
	T Allele frequency	76 (49)	40 (72.7)	(OR = 2.772) (95% CI: 1.416 to 5.428) (p = 0.004)	
rs2228570 (VDR)	TT	21 (13.5)	4 (7.3)	(χ^2^ = 1.651) (p = 0.438)	
	CC	75 (48.4)	30 (54.5)		
	TC	59 (38.1)	21 (38.2)		
	C Allele frequency	134 (86.5)	51 (92.7)	OR = 1.998 (95% CI: 0.6538 to 6.106) (p =0.321)	
rs731236 (VDR)	TT	48 (31)	18 (32.7)	(χ^2^ = 0.058) (p = 0.971)	
	CC	26 (16.8)	9 (16.4)		
	TC	81 (52.3)	28 (50.9)		
	C Allele frequency	107 (69)	37 (67.3)	OR = 0.922(95% CI: 0.4774 to 1.781)(p = 0.942)	

The genotype frequencies of *rs1143634* of the *IL-1β* gene were inversely associated with IDD. The frequency of T allele was almost 1.5 folds higher in control group (72.7% versus 49%, p=0.004, OR= 2.772, CI: 1.416 to 5.428). Additionally, this was reflected in genotype frequencies observed in the control group – 51% versus 27.3% for CC; 11.6% versus 1.8% for TT; and 37.4% versus 70.9% for CT, respectively (p<0.0001) (Table 3). Additionally, frequencies of T allele in patients and control were 49% versus 72.7% (p=0.004; OR= 2.772; 95% CI: 1.416to 5.428). Therefore, our results demonstrated that the presence of *rs1143634* polymorphism of *IL-1β* (T allele) was higher in controls than in patients, indicating no association between this polymorphism and IDD; also, CC genotype was more frequent in patients in comparison to the control group (Table 3).

Allelic frequencies of *rs2228570* polymorphism of the *VDR* gene in patients and controls were as the following: C allele 86.5% in patients versus 92.7% in the control group (p=0.438; OR=1.998; 95% CI: 0.6538 to 6.106). Genotypes frequencies were 13.5% versus 7.3% for TT; 48.4% versus 54.5% for CC; and 38.1% versus 38.2% for TC, in patients and control group, respectively (p=0.438)(Table 3). Our findings showed that the TT genotype was predominant in patients (1.998 fold higher) (Table 3). For *rs731236* polymorphism of *VDR* gene, the genotypes frequencies among patients and controls were 31% versus 32.7% for TT; 16.8% versus 16.4% for CC; and 52.3% versus 50.9% for TC, respectively (p=0.971) (Table 3). The distribution of C allele was 69% versus 67.3% (p=0.942; OR=0.922; 95% CI: 0.4774to 1.781), respectively. Thus there was no significant association between this polymorphism and IDD disease (p>0.05) (Table 3).

The analysis of the association among the studied polymorphisms and IDD according to sex revealed that there was no significant association between any of the polymorphisms and IDD (Table 4 and Table 5).

**Table 4:** Genotypes and allelic frequencies of the polymorphisms rs1800587 of IL-1α gene, rs1143634 of IL-1β gene, and rs2228570 and rs731236 of VDR genes in females for patients and controls

	Genotype	Patients (%)	Control (%)	Statistical analysis	
rs1800587 (IL-1α)	CC	35 (42.7)	13 (41.9)	(χ2 1.172) (p = 0.557)	
	TT	14 (17.1)	3 (9.7)		
	CT	33 (40.2)	15 (48.4)		
	T Allele frequency	47 (57.3)	18 (58.1)	(OR = 1.031)(95% CI: 0.4464 to 2.381)(*p* = 0.943)	
rs1143634 (IL-1β)	CC	43 (52.4)	8 (25.8)	(χ2 = 11.712) (p = 0.003)	
	TT	10 (12.2)	1 (3.2)		
	CT	29 (35.4)	22 (71)		
	T Allele frequency	39 (47.6)	23 (74.2)	(OR = 3.17)(95% CI: 1.271 to 7.907)(*p* = 0.02)	
rs2228570 (VDR)	TT	17 (20.7)	2 (6.5)	(χ2 = 5.36) (p = 0.069)	
	CC	28 (34. 1)	17 (54.8)		
	TC	37 (45.1)	12 (38.7)		
	C Allele frequency	65 (79.3)	29 (93.5)	(OR = 3.792)(95% CI: 0.8216 to 17.505)(*p* = 0.126)	
rs731236 (VDR)	TT	26 (31.7)	8 (25.8)	(χ2 = 0.997) (p = 0.607)	
	CC	17 (20.7)	5 (16.1)		
	TC	39 (47.6)	18 (58.1)		
	C Allele frequency	56 (68.3)	23 (74.2)	(OR = 1.335)(95% CI: 0.5269 to 3.381)(*p* = 0.704)	

**Table 5:** Genotypes and allelic frequencies of the polymorphisms rs1800587 of IL-1α gene, rs1143634 of IL-1β gene, and rs2228570 and rs731236 of VDR genes in males for patients and controls

	Genotype	Patients (%)	Control (%)	Statistical analysis	
rs1800587 (IL-1α)	CC	32 (43.8)	11 (45.8)	(χ2 = 0.441) (*p* = 0.802)	
	TT	9 (12.3)	4 (16.7)		
	CT	32 (43.8)	9 (37.5)		
	T Allele frequency	41 (56.2)	13 (54.2)	(OR = .922)(95% CI: 0.3651 to 2.330)(p = 0.864)	
rs1143634 (IL-1β)	CC	36 (49.3)	7 (29.2)	(χ2 = 7.252) (*p *= 0.027)	
	TT	7 (9.6)	0 (0)		
	CT	30 (41.1)	17 (70.8)		
	T Allele frequency	37 (50.7)	17 (70.8)	(OR = 2.363)(95% CI: 0.8755 to 6.377)(*p *= 0.137)	
rs2228570 (VDR)	TT	7 (9.6)	2 (8.3)	(χ2 = 0.298) (p = 0.862)	
	CC	43 (58.9)	13 (54.2)		
	TC	23 (31.5)	9 (37.5)		
	C Allele frequency	6 (90.4)	22 (91.7)	(OR = 1.167)(95% CI: 0.2253 to 6.040)(p = 0.854)	
rs731236 (VDR)	TT	20 (27.4)	10 (41.7)	(χ2 = 2.282) (p = 0.320)	
	CC	10 (13.7)	4 (16.7)		
	TC	43 (58.9)	10 (41.7)		
	C Allele frequency	53 (72.6)	14 (58.3)	(OR = 0.528)(95% CI: 0.2021 to 1.381)(p = 0.290)	

## Discussion

The genetic predisposition has been studied for its contribution to the degeneration process leading to an acceleration of ECM degradation or influencing inflammation and pain [2] [3] [4] [8] [9] [11] [12] [13] [17] [18] [19]. Genes of interest include functional genes, such as *IL-1* gene and VDR, and structural genes, such as aggrecan and collagen. These sets of genes have been commonly studied due to their importance in normal mineralization and tissue remodelling [20] [21] [22] [23]. In this study, the association between the polymorphisms *rs1800587* of *IL-1α*, *rs1143634* of I*L-1β* and *rs2228570* and *rs731236* of *VDR*, and IDD has been investigated.

Previous studies suggested that *IL-1* participates in IVD degeneration through suppression of ECM proteoglycans and collagen synthesis, increasing the production of ECM-degrading enzymes by stimulating proteases synthesis [24]. Normally, *IL-1* is expressed and regulated in the disc cells through a balance of activating and inhibiting receptors (*IL-1RI* and *IL-1Ra*, respectively), in the case of disc degeneration, this process becomes unbalanced (local overproduction of *IL-1RI* and/or underproduction of *IL-1Ra*) [20]. The current study showed no significant association between *rs1800587* polymorphism of the *IL-1α* gene and IDD development, and some convincing evidence exists in the literature. For instance, a previous case-control study of the Spanish population found no association between T allele and symptomatic lumbar disc herniation [21]. Also, a study in the north-western Mexican Mestizo population showed that the distribution of T allele in patients and controls was 27.0% versus 28.0% (p=0.455), supporting the lack of association between *rs1800587* polymorphism and IDD [4]. Moreover, Kelempisioti et al. [25] demonstrated the lack of any correlation between *rs1800587* polymorphism and IDD. Interestingly, a significant association of *rs1800587* polymorphism with the IDD in a Finnish population exhibiting the TT genotype of the *IL-1α* gene as an increased risk factor of the disc bulges 3-folds compared to the CC genotype has been reported [26]. Another study on 12 to 14-year-old Danish children also found an association between *rs1800587* polymorphism and disc degeneration in females [27]. The inconsistent association between *rs1800587* polymorphism and IDD could be attributed to ethnic differences, where different haplotypes in the promoter or enhancer regions and/or environmental factors can explain the variations [4].

*IL-1β* has numerous pro-inflammatory properties that have been correlated with the pathogenesis of IDD [22]. The current study demonstrated that the presence of *rs1143634* polymorphism of *IL-1β *(T allele) was higher in the control group than in the IDD group. This finding can be related to the fact that IDD can be asymptomatic [28]. The control group in this study was chosen based on the absence of clinical symptoms only. However, an MRI scan, the gold standard for the diagnosis of IDD [29], could have revealed IDD in the control group, indicating the lack of association between this polymorphism and IDD. Videman et al [10], in a study on 588 Finnish men, have shown that *rs1143634* polymorphism is not associated with IDD in MRI diagnosed patients. Also, Karppinen et al. [30] reported that in a Finnish male population, the *rs1143634* polymorphism was not correlated with Modic changes in vertebral endplates. In addition, a study by Kelempisioti et al [25]. on 538 Finnish young adults did not find any correlation between *rs1143634* polymorphism and IDD. On the other hand, several studies found an association between *rs1143634* polymorphism and IDD, for instance, in Finnish individuals, the T allele of the *rs1143634* polymorphism of *IL-1* gene is more frequent in patients with disc bulge [26] [31]. These inconsistencies are explained by the interference of other genetic variations or environmental factors. Furthermore, Solovieva et al [32], in 2006, reported an interaction between the *rs1143634* polymorphism and the *Trp3* allele of the *COL9A3* gene. They showed that the carriage of the Trp3 allele of *COL9A3* in the absence of the T allele of *IL-1* increased the risk of dark nucleus pulposus and joint occurrence of degenerative changes, while there was no effect of the *Trp3* allele in the presence of the *IL-1* T allele, suggesting that *IL-1* gene polymorphism may modify the effect of the *COL9A3* in IDD patients [32].

Our results of *rs1800587* of *IL-1α* and *rs1143634* of *IL-1* polymorphisms, consistent with the findings of Noponen-Hietala et al. [16] did not find an association between *rs1800587* of *IL-1α* and *rs1143634* of *IL-1* polymorphisms and IDD. Therefore, other interleukin genes such as *IL-1RN* and their structural counterparts (e.g., Col9A3) should be taken into account, and their role in the IDD development investigated in this cohort.

The role of VDR gene polymorphism (*rs2228570*) was also examined in this cohort. Despite the high frequency of the TT genotype in the patients' group versus the control group. Our results did not show any significant association between *rs2228570* and the presence of IDD. Likewise, Eskola, in 2012, found no association between *rs2228570* polymorphism and IDD in young Danish females [31]. Also, Serrano et al. [4], in 2014, showed that *rs2228570* polymorphism of the DR gene was not associated with IDD. On the contrary, other studies have shown an association between *rs2228570* polymorphism and IDD [17]. *VDR* is involved in bone and cartilage maintenance, such that alterations in the ECM function and the sulfation of glycosaminoglycans affect the disc and can cause degeneration. The first reported involvement of *VDR* variants in IDD was in the Finnish population [17], and later on, in other studies in Japanese and Chinese populations, the association was demonstrated [11] [12]. On the other hand, many studies on genetically different populations have not found a relationship between *VDR* polymorphisms and IDD [31]. *Rs2228570* polymorphism (ATG to ACG) of *VDR* gene occurs at the first of the two potential translation initiation site in exon 2 in the 5' promoter region, individuals with the T allele begin translation at the first initiation site generating a complete protein, while those with the C allele start translation at the second initiation site and generate a smaller protein. The shorter allele interacts efficiently with the transcriptional factor and displays a slight increase in function when compared to the longer one.

While *rs731236* polymorphism is a single base substitution T/C (ATT to ATC) causing a synonymous change (isoleucine) at codon 352 in exon 9 of the *VDR* gene close to the 3' terminus, this polymorphism does not determine a structural modification in the receptor and is associated with the increased transcriptional activity, regulation of mRNA stability, and high serum level of 1,25-dihydroxy vitamin D3 [19]. However, our findings demonstrated that *rs731236* polymorphism of the VDR gene was not associated with IDD, on the contrary, a report of the Finnish population, Videman et al. found that the signal intensities of thoracic and lumbar (T6-S1) discs were 12.9% worse in men with the CC genotype and 4.5% worse in men with the TC genotype, compared with signal intensity in men with the TT genotype [17]. An association between *rs731236* and *rs2228570* polymorphisms of the VDR gene and IDD was reported in other populations like Japanese, Chinese, and Turkish [11] [12] [23].

Although the genetic basis of IDD is an area of current investigation that may define or lead to prevention or possible treatment options for disc degeneration, age-related and environmental factors which may synergistically culminate in disc degeneration should not be underestimated. This study has focused on obesity, where the mean BMI of the patient's group (31.0 kg/m^2^) was significantly higher than that of the control group (26.5 kg/m^2^). This increase in BMI among patients represents a proportional relationship between obesity and IDD, presumably as a result of increasing load on a vertebral disc [33] [34]. Additionally, the peptide hormone, leptin, secreted primarily from adipose tissues and is a marker of obesity, is also secreted from cartilage tissue cells, including nucleus pulposus (NP) cells of intervertebral discs. Leptin is capable of inducing the abnormal proliferation of NP cells, which might be a possible mechanism underlying the impact of obesity in disc degeneration [35] [36] [37].

Although the genetics of IDD needs to be further investigated, and eventually a GWAS type of studies must be launched as well, we should not underrate the modifiable and unmodifiable environmental factors such as obesity, occupation, smoking and biochemical stress (inflammation and oxidative stress) all in conjunction with the normal process of degeneration.

## Conclusion

This study demonstrated that polymorphisms of *rs1800587* of *IL-1α*, rs1143634 of *IL-1β*, and *rs2228570* and *rs731236* of *VDR* genes are not associated with IDD. Other factors, such as obesity, or gene-gene, gene-environment, or gene-age interactions, as illustrated by BMI, could potentially play a role. Further studies are needed to elucidate the potential association of other candidate genes polymorphisms and IDD in the Jordanian population. Understanding the etiology underlying IDD may help suggest a possible approach to delay the onset of IDD or, at least, aid in predicting the risk of developing the disease.

## Acknowledgements

This research was supported by the Deanship of Scientific Research and Graduate Studies at Yarmouk University, Grant #20/2017.

## Conflict of interest statement

The authors stated that they have no conflicts of interest regarding the publication of this article.
